# Effect of Different Downward Loads on Canal Centering Ability, Vertical Force, and Torque Generation during Nickel–Titanium Rotary Instrumentation

**DOI:** 10.3390/ma15082724

**Published:** 2022-04-07

**Authors:** Keiichiro Maki, Arata Ebihara, Hayate Unno, Satoshi Omori, Taro Nakatsukasa, Shunsuke Kimura, Takashi Okiji

**Affiliations:** Department of Pulp Biology and Endodontics, Division of Oral Health Sciences, Graduate School of Medical and Dental Sciences, Tokyo Medical and Dental University (TMDU), 1-5-45 Yushima, Bunkyo-ku, Tokyo 113-8549, Japan; a.ebihara.endo@tmd.ac.jp (A.E.); h.unno.endo@tmd.ac.jp (H.U.); s.omori.endo@tmd.ac.jp (S.O.); t.nakatsukasa.endo@tmd.ac.jp (T.N.); s.kimura.endo@tmd.ac.jp (S.K.); t.okiji.endo@tmd.ac.jp (T.O.)

**Keywords:** ProTaper NEXT, downward load, canal centering ratio, root canal instrumentation, vertical force, torque, instrumentation time

## Abstract

This study aimed to examine how downward loads influence the torque/force and shaping outcome of ProTaper NEXT (PTN) rotary instrumentation. PTN X1, X2, and X3 were used to prepare J-shaped resin canals employing a load-controlled automated instrumentation and torque/force measuring device. Depending on the torque values, the handpiece was programmed to move as follows: up and down; downward at a preset downward load of 1 N, 2 N or 3 N (Group 1N, 2N, and 3N, respectively; each *n* = 10); or upward. The torque/force values and instrumentation time were recorded, and the canal centering ratio was calculated. The results were analyzed using a two-way or one-way analysis of variance and the Tukey test (α = 0.05). At the apex level, Group 3N exhibited the least canal deviation among the three groups (*p* < 0.05). The downward force was Group 3N > Group 2N > Group 1N (*p* < 0.05). The upward force, representing the screw-in force, was Group 3N > Group 1N (*p* < 0.05). The total instrumentation time was Group 1N > Group 3N (*p* < 0.05). In conclusion, increasing the downward load during PTN rotary instrumentation improved the canal centering ability, reduced the instrumentation time, and increased the upward force.

## 1. Introduction

Root canal instrumentation that facilitates effective disinfection is a key objective of root canal therapy [1]. However, curved and constricted root canals pose the risk of creating iatrogenic aberrancies, such as ledges, apical canal deviations, and canal wall perforations, which may jeopardize the outcome of root canal therapy [2,3]. Nickel–titanium (NiTi) engine-driven instruments have become a widespread use since they are more flexible [4], maintain the canal curvature better [5], and offer a more favorable treatment outcome [6] than stainless steel hand instruments. However, the unexpected intracanal separation of rotating instruments is still a major concern in NiTi rotary instrumentation [7,8].

Proper manipulation of NiTi rotary instruments is important to preventing iatrogenic errors [8]. Several studies have focused on how the dynamics of the use of NiTi instruments influence the risk of intracanal instrument separation, and have identified factors that reduce the torque and/or force generation, including a shorter pecking depth [9], reciprocating motion (versus continuous rotation) [10], higher rotational speed [11,12], and higher pecking speed [13]. These factors may contribute to the reduction in stress accumulated in the rotating instruments, leading to a reduced risk of intracanal instrument separation.

The downward load applied to NiTi rotating instruments may be a factor that impacts stress generation [9]. Although gentle apical pressure is widely recommended [8], the magnitude of the downward load may largely depend on several operational factors related to the clinician’s handling behavior; thus, there is a lack of objective standardization [14]. Limited information is available on how the downward load applied to NiTi rotary instruments affects their shaping ability, stress generation, and shaping efficiency [14].

The aim of this study was to examine how different downward loads influence the canal centering ratio, torque/force development, and instrumentation time of ProTaper Next rotary instruments (PTN: Dentsply Sirona, Ballaigues, Switzerland), employing load-controlled automated root canal instrumentation. The null hypothesis was that the downward load did not influence the canal centering ratio, torque/force development, or instrumentation time when PTN was used for the instrumentation of curved root canals.

## 2. Materials and Methods

### 2.1. Sample Size Estimation

G*Power software (version 3.1.9.2, Heinrich Heine University, Düsseldorf, Germany) was employed at the effect size, α error and power of 1.4, 0.05 and 0.80, respectively, based on the data from preliminary experiments. The sample size was estimated as 10 in a group.

### 2.2. Downward Load-Controlled Root Canal Instrumentation and Torque/Force Measurement

**Figure 1:**
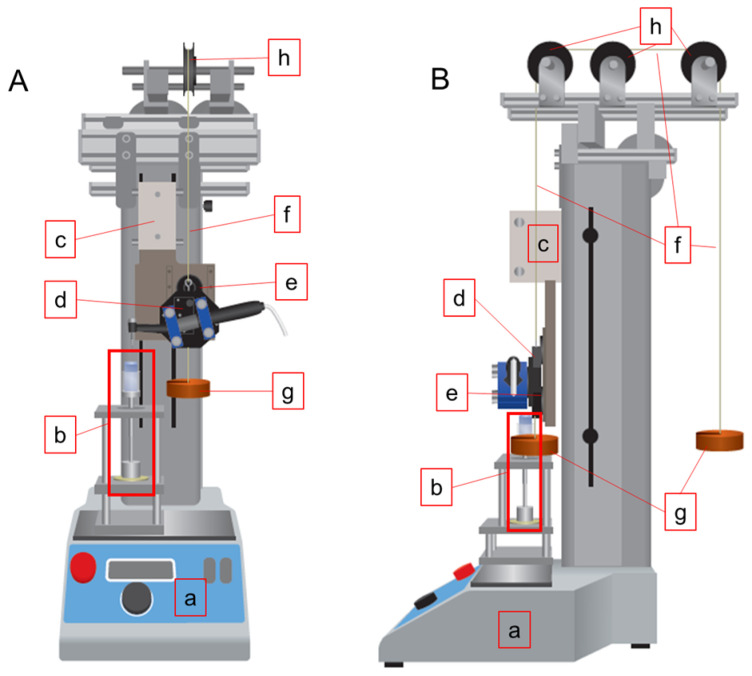
Schematics of the downward load-controlled root canal instrumentation device. (**A**) front view; (**B**) side view. a, motor-driven testing stand; b, torque/force measuring unit; c, mobile stage; d, handpiece holder; e, electromagnet; f, wire; g, weight; h, pulley.

A root canal instrumentation device comprising a low-speed, torque-controlled motor (J Morita, Kyoto, Japan) and a motor-driven testing stand (MX2-500N; Imada, Toyohashi, Japan) [13,15] was modified and used to control the magnitude of the downward load (Figure 1). A custom-made handpiece holder was fixed to the mobile stage of the testing stand with an electromagnet. The handpiece holder was hung with a wire and balanced by weights that were hung on the opposite side of the handpiece via three pulleys.

When the electromagnet was turned On, the handpiece and stage were programmed to move together at a preset speed of 50 mm/min [13]. When the electromagnet was turned Off, the handpiece was released from the stage and fell freely with a downward load controlled by the weights. To calibrate the downward load to 1 N, 2 N, and 3 N, vertical force values were measured when the electromagnet was turned Off and the head of the handpiece without a NiTi instrument directly touching the top of the canal model attached to the torque/force measuring unit.

The handpiece was programmed to make three types of movements depending on clockwise torque values detected by the motor as follows.

Movement 1: When the torque value was less than 0.2 N·cm, the electromagnet was programmed to be On, and the handpiece and stage made a downward movement for 2 s and an upward movement for 1 s at a speed of 50 mm/min [13]. The instrumentation always started with this movement.

Movement 2: When the torque value was between 0.2 N·cm and 2.5 N·cm, the electromagnet was programmed to be Off, and the handpiece fell freely with a preset downward load (1 N, 2 N, or 3 N).

Movement 3: When the torque value was more than 2.5 N·cm, the electromagnet was programmed to be On, and the handpiece and moving stage moved up together for 3 s at 50 mm/min. After moving up, the handpiece made one of the three types of movements depending on the torque measured by the motor.

A resin block having a J-shaped simulated canal (size #15, 0.02 taper, 45° curvature, 17 mm length; Endo Training Bloc, Dentsply Sirona) was fixed on a metal stage linked to the torque/force measuring unit with a metal rod. The measuring unit consisted of strain gauges (KFG-2-120-D31-11, Kyowa Electronic Instruments, Tokyo, Japan) and a load cell (LUX-B-ID; Kyowa Electronic Instruments), which were used for measuring the torque and force, respectively [13,15]. The output signals were amplified using an amplifier (PCD-400A, Kyowa Electronic Instruments) and transferred to a computer with data recording software (DCS-100A; Kyowa Electronic Instruments).

### 2.3. Root Canal Instrumentation

The resin blocks (*n* = 30) were instrumented with the full working length set to the apex. The ProTaper Gold SX instrument (Dentsply Sirona) was first used to flare the canal to 5 mm from the apex, and the ProGlider instrument (Dentsply Sirona) attached to the automated instrumentation device was used to establish a glide path to the apex. The resin blocks were then assigned randomly into Groups 1N, 2N, and 3N (each *n* = 10), in which the downward load was set at 1 N, 2 N, and 3 N, respectively. 

Each canal was instrumented with the PTN using the downward load-controlled automated root canal instrumentation device. X1 (size 17/0.04 taper at the tip area), X2 (size 25/0.06 taper at the tip area), and X3 (size 30/0.07 taper at the tip area) instruments were sequentially used. In each instrument, the instrumentation had two steps, i.e., to 1 mm short of the apex and then to the apex. A lubricating paste (RC Prep, Premier, Plymouth Meeting, PA, USA) was used during instrumentation. Following each use of the instrument, canal irrigation with 1 mL distilled water followed by patency verification using a size 10 K-file was performed. PTN instruments were used in one canal. 

During the PTN rotary instrumentation, the upward and downward force values and clockwise torque values were recorded, and the maximum values developed in each instrument were determined.

### 2.4. Evaluation of the Canal Centering Ratio and Instrumentation Time

**Figure 2:**
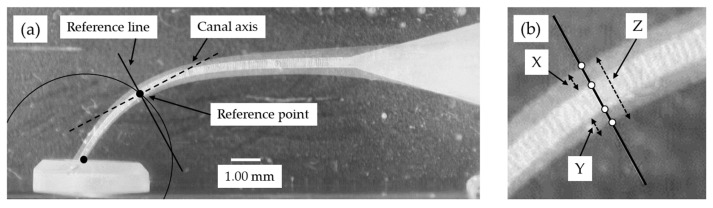
The method for measuring the amount of material removal. Photoshop software was used to superimpose images obtained before and after instrumentation. (**a**) A reference line is drawn by the determination of a reference point where the pre-instrumentation canal axis crosses a circle (0, 0.5, 1, 2, or 3 mm in diameter, corresponding to each measuring level) centering the pre-instrumentation canal apex; followed by the determination of a reference line as the line orthogonal to the canal axis passing through the reference point. (**b**) Four points are set at the intersections of the reference line and the pre- and post-instrumentation canal contours to measure the values corresponding to X, Y, and Z (see text above).

Image analyzing software (Photoshop 7.0, Adobe Systems, San Jose, CA, USA) was used to determine the canal centering ratio, as described previously [13,16]. Briefly, superimposed pre- and post-operative digital images were created (Figure 2), and the amount of material removed from the outer and inner canal wall was measured at five measuring levels (0, 0.5, 1, 2 and 3 mm from the apical terminus). The canal centering ratio was determined with the formula: (X − Y)/Z

where:

X = amount of material removed from the outer wall
Y = amount of material removed from the inner wall
Z = post-operative diameter of the canal.

The instrumentation time was defined as the time elapsed from the time point at which the torque value first exceeded 0.2 N·cm to the end of instrumentation. The time was calculated from the raw data of torque values acquired from the torque/force measuring unit.

### 2.5. Statistical Analysis

The normality and the homogeneity of the variance of the data were confirmed with the Shapiro–Wilk test and the Levene’s test, respectively. The canal centering ratio, the vertical force and clockwise torque values, and the instrumentation time were analyzed with a two-way analysis of variance followed by the Tukey test. A one-way analysis of variance and the Tukey test were used to analyze the total instrumentation time. The *p* value was considered to be significant at 5%.

## 3. Results

No instrument separation, distortion, or ledge formation was reported in any of the groups.

### 3.1. Canal Centering Ratio

**Figure 3:**
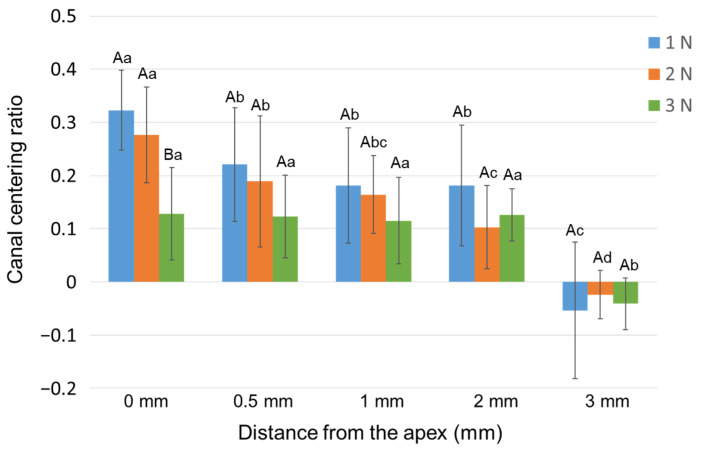
Effect of different downward loads on the canal centering ratio. Data are the mean and standard deviation (*n* = 10 in each group). Values with different capital letters at the same distance and values with different small letters at the same downward load are significantly different (*p* < 0.05).

The mean values of the canal centering ratio with three different downward load values are shown in Figure 3. At 0 mm from the apex, Group 3N showed the lowest centering ratio among all the groups (i.e., least deviation, *p* < 0.05). In Groups 1N and 2N, the absolute value at 0 mm was significantly greater than the values at all other measuring points (*p* < 0.05). 

### 3.2. Vertical Force and Clockwise Torque

**Figure 4:**
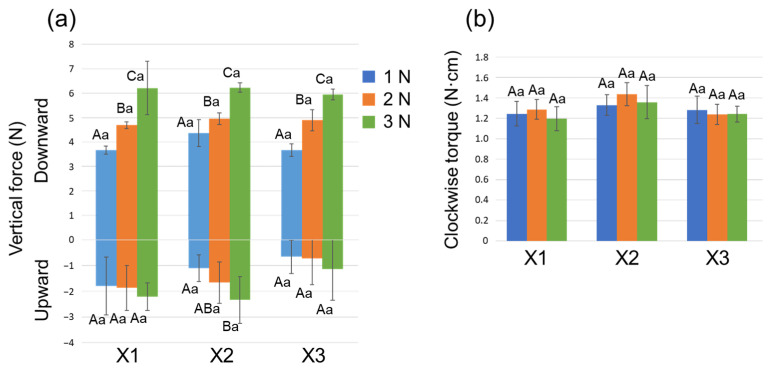
(**a**) Maximum upward and downward vertical force values and (**b**) maximum torque values detected during PTN instrumentation (X1, X2 followed by X3) with different downward loads. Data are the mean and standard deviation (*n* = 10 in each group for each instrument). Values with different capital letters in the same instrument and values with different small letters at the same downward load are significantly different (*p* < 0.05).

The mean maximum vertical force values and clockwise torque values developed during X1, X2 and X3 instrumentation with three downward loads are shown in Figure 4. Regarding the downward vertical force generated by the three instruments, Group 3N showed the largest force, followed by Groups 2N and 1N (*p* < 0.05). Regarding the upward vertical force, Group 3N recorded a significantly larger force than Group 1N for the X2-instrumentation (*p* < 0.05). The clockwise torque values showed no significant difference among the three instruments (*p* > 0.05). 

With the same instrument, different capital letters indicate significant differences (*p* < 0.05). With the same downward load, different small letters indicate significant differences (*p* < 0.05).

### 3.3. Instrumentation Time

**Figure 5:**
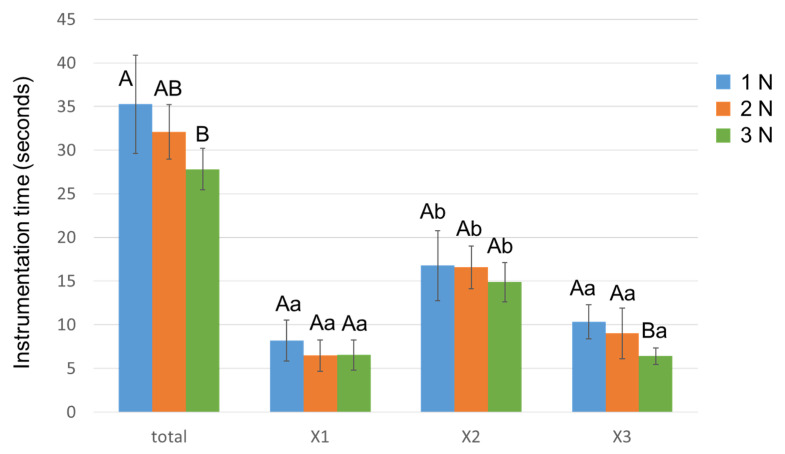
Time required for root canal instrumentation with different downward loads. The instrumentation time was measured for PTN X1, X2, and X3 instruments. Data are the mean and standard deviation (*n* = 10 in each group for each instrument). Values with different capital letters in the same instrument and values with different small letters at the same downward load are significantly different (*p* < 0.05).

Figure 5 shows the time required for total instrumentation and for each of the three instruments. The total instrumentation time was significantly shorter in Group 3N than Group 1N (*p* < 0.05). Group 3N recorded the shortest time for X3 instrumentation out of all the groups (*p* < 0.05). X2 exhibited the longest time among the three instruments in all groups (*p* < 0.05). 

## 4. Discussion

The dynamic torque and force characteristics of NiTi instruments during rotary root canal instrumentation have been investigated in numerous studies to assess the impact of various factors on the stress developed within the rotary instruments and the canal wall [14,17]. Thus, in addition to instrument-related factors, such as configuration and metallurgy [18], several operational factors that influence the stress development and shaping performance of NiTi rotary instrumentation have been identified [9,10,11,12,13]. Such factors that reduce torque and force generation and apical canal transportation include reciprocating motion [10] and a faster rotational speed [11,12]. Regarding the impact of factors related to the clinician’s handling behavior, a shorter pecking depth decreases the screw-in force [9]. Additionally, faster pecking speeds produce less apical canal transportation and more torque and apical force [13]. Few studies are available concerning the influence of the downward load, another important handling-related factor, on the stress development and shaping performance of NiTi rotary instrumentation. The current findings demonstrated that a larger downward load reduced the instrumentation time and degree of transportation, while a larger load produced a larger upward vertical force, which represented the screw-in force [19]. Thus, the null hypothesis was rejected.

Automated root canal instrumentation was previously employed to study the torque and force development and canal shaping performance of NiTi rotary instruments under strictly controlled laboratory conditions by excluding the influence of operator bias inherent in hand motion [20]. This study used a load-controlled automated root canal instrumentation device that was designed to apply pre-set downward loads to the rotating instrument when the torque value did not exceed the predetermined torque limit value. The axial movement was configured to simulate the motion that is applied clinically during NiTi rotary instrumentation: a pecking motion as the primary up and down motion and a withdrawing motion when an instrument meets resistance. The downward load values of 1 N, 2 N, and 3 N were determined from a preliminary study, where downward loads created by experienced operators during NiTi rotary instrumentation were monitored using the torque/force measuring unit, and average downward loads of 1.5–2 N were obtained. The load-controlled automated instrumentation excludes the influence of operator bias and may greatly contribute to determining the effect of downward load values on the preparation outcome under highly standardized conditions. However, the movement of the automated device may not fully reproduce an actual clinical hand motion, in which downward loads may be more variable [21]. Thus, care should be taken in extrapolating the present findings to a clinical situation. 

Simulated resin canals were used in this study to standardize the analysis by excluding anatomical variables and variations in the canal wall hardness inherent in natural teeth. Thus, various studies have employed resin canals instead of natural teeth to investigate torque/force generation [9,11,13,15,20] and apical canal transportation [22,23,24] during NiTi rotary root canal instrumentation. However, less force may be required to cut resin canals as they are softer with a smoother surface texture than dentin [25]. Additionally, the resin chips are larger than natural dentin chips, causing a more frequent canal blockage, particularly in the apical area [25]. These limitations may be compensated by using extracted teeth following the anatomical matching of the canals with micro-computed tomography [26].

The downward vertical force was significantly larger with an increasing pre-set downward load, indicating that the load application was conducted in a well-controlled manner. No significant difference in the clockwise torque was noted in all groups for each instrument size. The torque-limit setting and programmed movement of the handpiece, including the pecking speed [13], were appropriately adjusted so that the instruments required similar torque values during the shaping procedure irrespective of the different applied downward loads [14]. Thus, under the present conditions, the difference in the downward load can be assumed to be a factor closely associated with the observed differences among groups rather than the resulting clockwise torque generation.

PTN instruments were employed as a representative single-length rotary multiple-file system in which an improved cyclic fatigue resistance [27] and reduced tendency for apical transportation [28] were demonstrated compared with those of several other NiTi instruments. Such properties of the PTN instruments may be attributed to the use of a heat-treated M-Wire alloy [29] and its variable-tapered blade with an off-centered rectangular cross-section [27]. The peak vertical force generated by PTN is smaller than that by ProTaper Universal (Dentsply Sirona; manufactured from the conventional NiTi alloy); however, it is larger than that by Twisted File Adaptive (SybronEndo, Orange, CA, USA; manufactured from a heat-treated R-phase alloy) [30]. PTN develops greater torque generation than the ProTaper Universal [31] and Twisted File Adaptive [32]. These findings are important because several instrument-related factors, including the metallurgy and configuration of the NiTi instruments, influence the torque and force generation during instrumentation [14,17]. Thus, whether different NiTi rotary systems would perform differently under the present experimental conditions deserves further study.

The post-instrumentation root canals in all groups showed apical canal transportation to the outer wall, potentially because of the ability of NiTi instruments to recover their original linear shape during the instrumentation of the canal curvature [33]. Among operational and handling-related factors, the reciprocating motion (vs. continuous rotation) [10] and faster pecking speed [13] contribute to reducing the degree of apical transportation. In this study, Group 3N showed the least deviation 0 mm from the apex, which is attributed to the shorter instrumentation time of Group 3N. A shorter instrumentation time improves the canal centering ability, likely because of the reduced contact time of the blades with the canal wall, resulting in the removal of less resin from the outer canal wall [13].

These findings indicate that a larger downward load is associated with a larger screw-in force, which is represented by the upward vertical force acting on a canal model [15,19]. The screw-in force may induce instantaneous binding of a NiTi instrument, which exposes the instrument to a risk of breakage resulting from sudden and abrupt torsional stress [19]. The magnitude of the screw-in force varies depending on several factors including the taper, tip size, and pitch length of the instruments [9,19], heat treatment [16], kinematic motion (continuous vs. reciprocating motion) [15,34], and pecking depth [9]. The present findings suggest that a larger downward load should be considered a factor that augments the screw-in force. Because the screw-in force is closely associated with instrument engagement to the root dentin, it is reasonable that the large downward load could lead to strong binding of an instrument to the canal wall.

From a clinical perspective, knowledge on how the downward load influences the preparation behavior of NiTi rotary instruments is important to ensure the safe and efficient use of these instruments. The current study’s findings indicate that the downward load should be regarded as a significant factor that may affect the screw-in force-induced stress generation. Increasing the downward load may be beneficial to improving the shaping ability and reducing the preparation time of PTN rotary instrumentation, as long as the torque generation is appropriately controlled and the operator has sufficient skills and experience to manage the screw-in force. Additional studies using different brands of NiTi rotatory instruments on anatomically-matched extracted human teeth and adopting an automated handpiece movement that better mimics actual hand motion will deepen our knowledge on the effect of different downward loads on the shaping behavior of NiTi rotatory instrument systems.

## 5. Conclusions

The study findings revealed that increasing the downward load during PTN rotary instrumentation improved the canal centering ability, reduced the instrumentation time, and increased the upward force without increasing the clockwise torque.

## Data Availability

The data presented in this study are available on request from the corresponding author.
